# Building public trust and confidence in secondary use of health data for healthcare improvement and research: a qualitative study pre-protocol

**DOI:** 10.12688/hrbopenres.13711.1

**Published:** 2023-09-11

**Authors:** Tina Bedenik, Caitriona Cahir, K. Bennett

**Affiliations:** 1 School of Population Health, RCSI University of Medicine and Health Sciences, Dublin, D02 DH60, Ireland; 2 Science Foundation Ireland (SFI) FutureNeuro Research Centre, Dublin, D02 YN77, Ireland

**Keywords:** Qualitative research, health data, secondary use, trust, public and patient involvement, eHealth

## Abstract

**Background**

Secondary use of health data provides opportunities to drive improvements in healthcare provision, personalised medicine, comparative effectiveness research, health services innovation, and policy and practice. However, secondary data use requires compliance with relevant legislation, implementation of technical safeguards, ethical data management, and respect for data sharers. Existing evidence suggests widespread support for secondary use of health data among the public, which co-exists with concerns about privacy, confidentiality and misuse of data. Balancing the protection of individuals’ rights against the use of their health data for societal benefits is of vital importance, and trust underpins this process. The study protocol explores how to build public trust and confidence in the secondary use of health data through all key stakeholder groups in Ireland, towards developing a culture that promotes a safe and trustworthy use of data.

**Methods**

This study will adopt a qualitative cross-sectional approach conducted in accordance with the Consolidated Criteria for Reporting Qualitative Research COREQ guidelines. Participants in the study will include academics and researchers; healthcare professionals, data protection, ethics and privacy experts and data controllers; pharmaceutical industry and patients and public. Purposive and convenience sampling techniques will be utilised to recruit the participants, and data will be collected utilizing focus groups that may be supplemented with semi-structured interviews. Data will be coded by themes using reflexive thematic analysis (TA) and collective intelligence (CI) will be convened post-analysis to explore the preliminary findings with the participants.

**Ethics and Dissemination**

Ethical approval was obtained from the Royal College of Surgeons in Ireland Research Ethics Committee (REC202208013). Final data analysis and dissemination is expected by Q1 2024. Findings will be disseminated through peer-reviewed journal publications, presentations at relevant conferences, and other academic, public and policy channels. Lay summaries will be designed for Public and Patient Involvement (PPI) contributors and general public.

## Introduction

The proliferation of digital health data and the increasing adoption of electronic health records (EHR) and electronic patient registers will allow interrogation of large volumes of individual or population-level health data for secondary purposes. Secondary use of health data can be defined as “ *any use of health data for reasons other than those for which they were collected in the first place*” ( Perrin & Mathieu, 2021), which is sometimes used interchangeably with the use of data for ‘purposes beyond the direct care’ of the patient, such as quality improvement, health service planning and research ( The Health Information and Quality Authority, 2021). Secondary use of health data can drive improvements in healthcare provision and health outcomes, personalised medicine, comparative effectiveness research, health services innovation and policy and practice. However, secondary use requires compliance with relevant legislation, implementation of appropriate policies, procedures and technical safeguards, safe and ethical data management, and importantly respect for the privacy and confidentiality rights of data subjects.

International evidence on the public attitudes towards the use or sharing of health data for secondary purposes has increased in recent years, with a focus on the general knowledge, opinions and attitudes, as well as benefits and concerns related to secondary data use. This includes both systematic reviews ( Aitken *et al*., 2016a; Aitken *et al*., 2016b; Garrison *et al*., 2016; Hill *et al*., 2013; Howe *et al*., 2018; Hutchings *et al*., 2020; Hutchings *et al*., 2021) and primary qualitative and mixed-methods studies ( Audrey *et al*., 2016; Cheah *et al*., 2018; Grant *et al*., 2013; Haddow *et al*., 2011; Hate *et al*., 2015; Hill *et al*., 2013; Papoutsi *et al*., 2015; Spencer *et al*., 2016; Stevenson *et al*., 2013). The existing evidence base suggests a widespread support for secondary use of health data amongst the public, which co-exists with concerns and fears about breaches of privacy, confidentiality, misuse of data, sharing data for profit, discrimination and stigma that may affect data sharers. Earlier research especially indicated a low level of understanding and a lack of knowledge amongst the public about the existing practices, security and safeguards, and uses of health data for secondary purposes, suggesting that public education and information provision may increase public’ willingness to share their health data for secondary purposes ( Aitken *et al*., 2016a; Carson *et al*., 2019; Haddow *et al*., 2011; Hill *et al*., 2013). Despite this, there is an identified widespread support amongst the public for using health data for secondary purposes, with altruism – also referred to as common, greater or social good – and a sense of moral responsibility reported as the central motives for sharing data ( Aitken *et al*., 2016a; Garrison *et al*., 2016; Howe *et al*., 2018; Hutchings *et al*., 2020; Hutchings *et al*., 2021; Kalkman *et al*., 2022; Platt & Kardia, 2015; Spencer *et al*., 2016). The evidence also suggests that the public’s willingness to share health data for secondary purposes is not contingent only upon the perceived actual or potential benefits resulting from data use, but also upon real or perceived concerns about risks and harms resulting from data use. This includes concerns about the security and privacy of data sharers, data sharing with commercial entities and pharmaceutical industries specifically, lack of transparency and accountability around data sharing practices, data falling into the ‘wrong’ hands such as marketing agencies, employers and banks, misuse of information, unwanted impact of data disclosure, and stigma and discrimination related to disclosure of health data and sensitive health data specifically ( Aitken *et al*., 2016a; Garrison *et al*., 2016; Hill *et al*., 2013; Howe *et al*., 2018; Kalkman *et al*., 2022; Papoutsi *et al*., 2015; Spencer *et al*., 2016; Stevenson *et al*., 2013). These multiple concerns around data sharing may be mitigated through enhanced individual control over who, how and which reasons uses one’s health data for secondary analysis ( Haddow *et al*., 2011; Kalkman *et al*., 2022; McGuire *et al*., 2008; Spencer *et al*., 2016), which is intricately tied with building and nurturing public trust in the individuals or organization that manage and use health data ( Aitken *et al*., 2016a; Baird *et al*., 2009; Carson *et al*., 2019; Howe *et al*., 2018; Hutchings *et al*., 2020; Papoutsi *et al*., 2015).

Empirical studies of the public attitudes towards the use of health data for secondary purposes in Ireland remain scant; however, the extant body of research supports the international evidence. A survey of public attitudes towards the use of health data from the general practitioners’ (GP) medical records found general support for the sharing of anonymous personal health data with researchers without the need for consent on individual study basis ( Buckley *et al*., 2011), and these findings were subsequently corroborated by a smaller qualitative study ( Clerkin *et al*., 2013). A more recent systematic review of the UK and Irish public attitudes towards use of health data for research identified widespread support for the sharing of health data for altruistic reasons, alongside concerns about patients’ privacy ( Stockdale *et al*., 2019). The willingness to share data was closely connected to public trust in the individual or the organisation that managed their data, and their perceived competence to keep the data safe. Trust also emerged as a major theme from a recent qualitative study that examined the factors that influenced the public’s willingness to share their health data ( Flaherty *et al*., 2022). The three identified factors that shaped participants’ level of trust were: (i) their ability to exercise control over personal data that they believed belongs to them, (ii) the perceived transparency of the data sharing processes, and (iii) the perceived confidentiality of the healthcare service. This study ( Flaherty *et al*., 2022) was part of a wider mixed methods study of national public engagement on health information carried out by the Health Information and Quality Authority (HIQA) in partnership with the Department of Health and the Health Service Executive (HSE) (2021), conducted following the Conti cyber attack ( PwC, 2021) on the HSE in Ireland. This mixed methods study ( HIQA, 2021) suggested that the Irish public is overwhelmingly in support of using health data for secondary purposes, upon the condition that appropriate safeguards are in place. The study also found that the public wanted to be informed about how their health data is collected, used and shared by healthcare professionals, and for which purposes. Similar attitudes were echoed in the verdict from a citizens’ jury on access to health information published by the Irish Platform for Patient Organisations, Science and Industry (IPPOSI) (2021). The results captured a belief that health data belongs to the citizens, who need to be able to individually and regularly decide on how their information is accessed, used and shared. Advocating for a model of consent that is co-designed with the citizen, the citizens’ jury emphasised the need to put the citizens in control of the management of their health data and build public trust regarding health data collection and use. 

With some exceptions ( Grant *et al*., 2013) both the international and national research rarely explored perspectives of patients and public regarding secondary use of health data in conjunction with key stakeholders, which indicates the need for this particular study. In addition, the forthcoming legislative and policy changes in the European Union and Ireland further underscore the need to understand and secure public support regarding secondary health data use. The draft *European Declaration on Digital Rights and Principles* (2022) recommended by the European Commission will serve as a guide for policy makers and companies when dealing with new technologies, whilst the proposed European Health Data Space (EHDS) will provide a health specific ecosystem that supports the free movement of health data for secondary purposes. In Ireland, the upcoming Health Information and Patient Safety Bill will support the ongoing eHealth initiatives such as rolling out the individual health identifier and electronic health records, leading to a radical transformation of the national health information services. Building on the body of research, and in light of these legislative changes, the aims of the proposed study are twofold: (i) to explore the rationale for and means of using secondary health data for healthcare improvement and research, and (ii) to explore how to develop a culture of trust that promotes safe use of health data. Specifically, the study will focus on the key stakeholders’ knowledge, experiences and attitudes related to secondary health data use; their perceptions of risks, benefits and ethical challenges; internal components and processes underpinning public trust; as well as barriers and enablers to building trust and confidence in using health data for healthcare improvement and research.

## Methods

The study will be conducted and reported in accordance with the Consolidated Criteria for Reporting Qualitative Research COREQ: 32-Item Checklist ( Tong *et al*., 2007) to ensure methodological rigor, transparency and credibility of the findings.

### Study design

This study adopts a qualitative cross-sectional approach utilizing focus groups and/or semi-structured interviews in exploring how to build public trust and confidence in the secondary use of health data for healthcare improvement and research in Ireland. A qualitative approach was chosen in order to elicit and interpret a diversity of views and perspectives, and to allow the participants the necessary time to engage with and reflect on the subject matter through facilitative questioning ( Ritchie & Lewis, 2003). The qualitative design has previously been used in studies of people’s attitudes regarding health data use and sharing ( Baird *et al*., 2009; Cheah *et al*., 2018; Evans *et al*., 2020; Grant *et al.*, 2013; Hate *et al*., 2015; Spencer *et al*., 2016; Stevenson *et al*., 2013). Previous qualitative studies of health data use and sharing have used focus groups ( Clerkin *et al*., 2013; Haddow *et al*., 2011; Hill *et al*., 2013), interviews ( Audrey *et al*., 2016) or alternatively a combination of different data collection techniques such as focus group and survey ( Papoutsi *et al*., 2015) and focus group and a workshop ( Aitken *et al*., 2016b). Qualitative data collection techniques are a common approach in researching people’s perspectives on health data use and sharing, and focus groups provide the opportunity to collect rich in-depth information in a less-intense environment ( Smithson, 2000; Then *et al*., 2014). 

### Stakeholders and Patient and Public Involvement (PPI)

In order to ensure that the study is aligned with the standards and strategies regarding secondary use of health data endorsed by the key organisations in Ireland, individual remote meetings with the Irish Platform for Patient Organisations, Science and Industry (IPPOSI), Health Information and Quality Authority (HIQA) and Health Service Executive (HSE) took place in November and December 2022. These meetings provided an opportunity to inform the respective organisations about the study, discuss the study design and receive suggestions on the additional topics and questions that may be explored, and to exchange knowledge and information regarding the changing national policy landscape on health data. A Patient and Public Involvement (PPI) Panel for health research was also involved in the design of this study. Two older male adults reviewed research materials including the Participant Information Leaflet, Consent Form and topic guide and they provided written feedback on these materials, which was followed up with a video-call with one panel member. The research materials were subsequently amended prior to submission to the RCSI Research Ethics Committee.

### Research team and reflexivity

To increase the rigor, clarity and transparency of the qualitative process ( Jootun *et al*., 2009; Probst, 2015) researchers’ respective professional backgrounds and roles are outlined. Tina Bedenik (TB) is a Postdoctoral Fellow in the RCSI University of Medicine and Health Sciences, School of Population Health. She has an interdisciplinary background focusing on the intersection of health and social justice, and she specialises in qualitative research and participatory methodologies. Caitriona Cahir **(**CC) is a Lecturer in the RCSI Data Science Centre in the School of Population Health with research interests in epidemiology and psychology. She has experience in both quantitative and qualitative methodologies, and she collaborates with a wide range of academic disciplines. Kathleen Bennett (KB) is an Associate Professor in Biostatistics and Head of the Data Science Centre in RCSI. She has over twenty-five years’ experience in undertaking medical research, mainly quantitative research as well as qualitative and mixed methods studies.

The focus groups and any semi-structured interviews will be organised and conducted by TB, and a support person will be present to take notes. Data analysis will be conducted by TB, with support from KB and CC. We are cognizant that our respective backgrounds, experiences and knowledge may influence the research process, in particular data collection and analysis. In order to reduce researcher bias, the research team will review emerging themes in a collaborative manner, explore differences in coding, and critically reflect on any bias that may occur in this process. This will subsequently strengthen the quality of the analysis, and increase trustworthiness and validity of the research findings.

### Participants and recruitment

Participants in this study will be clustered into five homogeneous groups: Study Group 1 (SG1): Academics and Researchers using secondary data for research; Study Group 2 (SG2): Healthcare Professionals from primary care and hospital settings; Study Group 3 (SG3): Data Protection, Ethics and Privacy Experts and Data Controllers involved in health data; Study Group 4 (SG4): Pharmaceutical Industry; Study Group 5 (SG5): Patients and Public. Participants will be selected based on the following inclusion criteria: belonging to one of the five identified groups, experience with secondary health data use (applicable to SG1-4), and an interest in participating in the exploration of this topic regardless of their prior knowledge of secondary health data use (SG5). The study will only include participants aged 18+ years who are able to provide written, informed consent. The study will exclude those who cannot communicate fluently in English language. Eligibility criteria was based on the literature review of qualitative and mixed methods studies ( Aitken *et al*., 2016b; Baird *et al*., 2009; Buckley *et al*., 2011; Cheah *et al*., 2018; Damschroder *et al*., 2007; Evans *et al*., 2020; Grant *et al*., 2013; Haddow *et al*., 2011; Hill *et al*., 2013; McGuire *et al*., 2008; Papoutsi *et al*., 2015; Spencer *et al*., 2016; Stevenson *et al*., 2013; Trinidad *et al*., 2010), and it was further informed by discussion within the research team and with the key stakeholders.

The aim is to facilitate one focus group for SG1-4 and up to four focus groups for SG5. It is anticipated that each focus group will consist of four-eight participants, and the proposed sample size will range between 36 and 64 participants. The sample size is based on the literature review of qualitative studies of health data use and sharing that utilised a combination of focus groups and interviews ( Baird *et al*., 2009; Cheah *et al*., 2018; Evans *et al*., 2020; Grant *et al* 2013; Hate *et al*., 2015; Spencer *et al*., 2016; Stevenson *et al*., 2013), a focus group and a workshop ( Aitken *et al*., 2016b) and just focus groups ( Clerkin *et al*., 2013; Haddow *et al*., 2011; Hill *et al*., 2013; McGuire *et al*., 2008; Trinidad *et al*., 2010). The sample size in the above cited studies ranges from fifteen participants ( McGuire *et al*., 2008) to sixty-six participants ( Hate *et al*., 2015), with the mean number across these thirteen qualitative studies being forty-five participants. However, it should be noted that some of these studies were multi-team and longer-lasting research projects. In addition, some challenges with organizing focus groups are anticipated, and they may need to be supplemented with semi-structured interviews. Therefore, the final sample size in this study will be contingent upon the success of the recruitment process, responsiveness of the invited individuals and resource availability. 

This project will utilise purposive and convenience sampling techniques, which is in line with other extant qualitative research on health data use and sharing ( Cheah *et al*., 2018; Grant *et al*., 2013; Hate *et al*., 2015; Spencer *et al*., 2016). Relevant representative bodies and organizations in Ireland will be approached in writing with a request to invite their members and/or representatives to take part in the study through their (social) media channels. In relation to recruitment for the SG5 (Patients and Public), non-governmental organisations that advocate for and support specific social groups, including disadvantaged and under-represented ones, will be approached. This is to ensure diversity of viewpoints and representation of different communities in this study, especially those that may have particular needs or concerns regarding access, use and sharing of individual health data. Specifically, focus will be on the organisations working in the following fields: mental health; reproductive and sexual health; gender and LGBTQI; disability; homelessness and addiction. We also aim to include representatives and/or service users of organizations that work with and support ethnic minorities, migrants and asylum seekers and the Travelling community. In addition, we will use advertisements on websites associated with the research area, social media (e.g. Twitter, Facebook), newsletters and research flyers to ensure that all representative groups are included. Some potential participants for SG1-4 have already been identified through participation in online and in-person seminars and workshops on health data in Ireland, such as Epilepsy Ireland National Conference 2022.

### Data collection

It is anticipated that between five and eight focus groups (four for SG1 to SG4, and up to four for SG5) will take place in early and mid-2023 online. There will be separate focus groups for different categories of participants, and each individual focus group will meet once. Participants who are unable to attend their assigned focus group, will be invited for an individual interview. The focus groups are expected to last between one-two hours and interviews around one hour. Once consent had been obtained, basic demographic data will be collected from all participants including information about their gender, age range, ethnicity, educational level and field of work (if applicable). This information will be presented in an aggregate form in order to provide context to the findings.

Both focus groups and/or interviews will be conducted using a topic guide. The topic guide for all the Study Groups will include questions pertaining to participants’ knowledge, experiences and attitudes related to secondary health data use; their perceptions of risks, benefits and ethical challenges regarding secondary health data use; internal components and processes underpinning public trust; and barriers and enablers to building trust and confidence in using health data for healthcare improvement and research. Not all questions in the topic guide will be relevant or applicable to all five Study Groups and the guide is tailored for each individual Study Group. The topic guide was informed by the literature review and it had been discussed with the RCSI PPI Panel, Industry Partner IQVIA Ireland and HIQA, IPPOSI and the HSE. The topic guide tailored for SG5 (Patients and Public) will be piloted in an online semi-structured interview with a female PPI Panel member, and the topic guide tailored for SG1-4 will be piloted in an online semi-structured interview with a qualitative researcher in the RCSI School of Population Health. The topic guide is flexible yet focused in its nature, which enables the research team to gather information that is relevant and adequate the purpose of the study, whilst keeping data collection at minimum

Cognizant that not all participants in SG5 (Patients and Public) may have prior knowledge about secondary use of health data, a short presentation at the start of this focus group will be facilitated in order to enable an informed discussion. It is anticipated that during focus groups some participants may respond to questions in a way that they believe is socially acceptable. In order to reduce the chance of participant bias, the research team will introduce open-ended question that cannot be addressed with a simple yes/no answer. Furthermore, questions may be asked in an indirect way to prevent from participants feeling pressured to respond, and prompts may be used to approach one subject from various angles. Specific emphasis will be placed on mutual respect and confidentiality, and participants will be explicitly asked not to talk about their own medical conditions.

Focus groups and/or interviews will be audio-recorded and video recorded. Video recordings, however, will be deleted immediately after data collection, and only audio recordings will be retained. All audio recordings will be sent to a transcribed by a third-party party transcription service for transcription, and subsequently shared with those participants who expressed their wish to receive them. Any changes that participants make pertaining to their own statements will be implemented, and the transcriptions will be de-identified and uploaded to NVivo 12 qualitative data analysis software to enable analysis. The original transcripts and audio recordings will be destroyed immediately following de-identification.

### Data analysis

Qualitative data will be transcribed verbatim, de-identified and imported into NVivo 12 software to organise the data and conduct analysis. The transcripts will be scrutinised and data will be coded by themes using an inductive and iterative approach. Commonly used in qualitative health research, reflexive thematic analysis (TA) ( Braun & Clarke, 2006; Braun & Clarke, 2019) is chosen for its flexible and adaptable approach that enables a production of a rich and detailed account of data. The analysis will be a data-driven process, which means that the research team will search for recurrent patterns of meanings from the data and generate themes, rather than using a pre-existing coding framework to guide their analysis ( Braun & Clarke, 2006; Braun & Clarke, 2019). Six phases of analysis ( Braun & Clarke, 2006; Braun & Clarke, 2019) will be used a guide to this process: familiarization with the data; generating initial codes; searching for themes; reviewing themes; defining and naming themes and producing the report. However, reflexive TA is not a linear process, and some movement back and forth between these six phases is anticipated. Adherence to the 15-Point Checklist of Criteria for Good Thematic Analysis ( Braun & Clarke, 2006) will support the methodological rigor during the analysis and writing up the research findings. The term reflexive TA captures the philosophical foundation upon which this method is based and highlights the crucial role that the researchers play in the process of knowledge production. This method replies upon an awareness that the coding process requires “a continual bending back on oneself” ( Braun & Clarke, 2019:6), which includes an interrogation of researchers’ assumptions that that shape this process. In light of that cognition, the two members of the research team will independently code two interviews at the start of the analysis, and subsequently compare and discuss results to develop a coding index. Finally, attention will be devoted to the researchers’ positionality in the attempt to identify and reduce any bias, and to enable a production of more trustworthy knowledge ( Nowell *et al*., 2017).

### Collective intelligence

Upon the completion of data analysis, the aim is to convene an assembly of all study participants to present the preliminary findings, and to explore these in a group setting. This participatory research method is knowns as collective intelligence (CI), which can be broadly defined as “groups of individuals doing things collectively that seem intelligent” ( Malone *et al*., 2009). This method enables interaction amongst a range of individuals in order to enable a production of shared insight and more accurate study outcomes ( Kurvers *et al*., 2015; Wolf *et al*., 2015). The underlying idea driving this approach is that the access to a wide social group, in which knowledge is distributed, implies access to wide epistemic resources ( Broadbent & Nicholas, 2015). In addition, research indicates that this CI may be increasingly employed because research problems are becoming more complex, and traditional research methods anchored in one discipline no longer fit researchers’ needs ( Nguyen *et al*., 2019). In recent years, this method has been gaining more traction in medical and healthcare research. For instance, a recent systematic scoping of studies utilising CI in medical decision-making revealed that CI helps resolve uncertainty in decision-making, it can improve metacognitive activities (awareness of one’s own thinking process) and collective insight, and thus results in more accurate outcomes ( Radcliffe *et al*., 2019). In addition, the CI approach was employed in a study of barriers and needs of adults using eHealth for chronic pain self-management ( O’Reilly *et al*., 2022), assessment of the citizens’ interpretation of public health information ( Guilbeault & Centola, 2020), exploration of challenges and barriers to accessing, understanding and using open data ( Hogan *et al*., 2017), and it was argued to be a valuable method in eliciting insights into the values and preferences of citizens regarding design of wellbeing measures and policies ( Hogan *et al*., 2015). Adoption of CI enables triangulation, increases credibility and validity of the research findings, and allows the research team to base the study recommendations on a group consensus.

## Ethics

Ethical approval for the qualitative study was obtained from the RCSI Research Ethics Committee (REC) on 8 November 2022. Interested individuals will receive the Participant Information Leaflet (PIL) and the Consent Form in an electronic form or hard copy, depending on the mode of data collection, prior to the focus groups/interviews taking place. A one-week time interval will be allowed between providing research materials and seeking consent, which provides the individuals with time to reflect on the study, consider their involvement and ask further questions. All participants will be required to sign the Consent Form in person or electronically prior to any data collection.

The participants may withdraw from the study at any stage up and up until they have the opportunity to review the transcript and make changes to their statements. Following data collection, the audio recordings of focus groups and interviews will be transcribed by an external service provider, with whom a data processing agreement has been put in place. Different mechanisms will be in place to protect participants’ privacy and confidentiality rights. Specifically, the transcriptions will be de-identified, and all names, locations and other potentially identifiable data will be replaced with number, letter or participant ID. De-identified transcripts will be uploaded to NVivo 12 software to enable data analysis, and the original transcripts and audio or video recordings will be deleted. The de-identified data will be stored on the RCSI OneDrive and password-protected computers, which are only accessible only to the research team. Upon the completion of the study, the de-identified transcripts of those participants who gave consent will be deposited with Zenodo, an open repository developed under the European OpenAIRE program that covers all types of research output including qualitative data.

All participants in this study will have their travel expenses reimbursed for travelling to RCSI for focus groups and/or interviews. Participants in the Patients and Public focus group will receive an honorarium (voucher) for their time participating in the focus group or interview, irrespective of whether in-person or virtual participation. This is standard practice for Public and Patient involvement in research ( www.ppinetwork.ie).

## Dissemination

Final data analysis and dissemination is expected by Q1 2024. The results will be written up in aggregated form for publication through a range of academic, public and policy channels including conferences, presentations, peer-reviewed journals, scientific reports, newsletters, websites, podcasts and social media, and lay summaries and infographics will be designed for general public. Those participants that expressed their interest in the final report will receive a copy upon study completion.

## Study status

At the time of publication, the research team is planning to pilot the topic guide and start with the recruitment in Q1 2023.

## Conclusion

The international and Irish literature and the upcoming legislative changes (The Health information and Patient Safety Bill and The European Health Data Space) demonstrate an increasing importance for the support for secondary use of health data amongst the public, and an awareness of benefits for sharing health data. Enhanced knowledge and awareness about this topic is positively associated with the willingness to share health data, and the public wishes to be informed about who is using their data, which is being data, and for what purpose. Nonetheless, concerns about sharing data remain widespread and these include fears about breaches of privacy, confidentiality and misuse of data and discrimination. In the light of these competing priorities, the central dilemma emerging from the evidence is how to balance the protection of individuals’ rights against the use of their health data for societal benefits. No qualitative study on secondary use of health data that explores the aforesaid issues and includes all relevant stakeholders and PPI has been conducted in Ireland to date. Encompassing the general public, patients, healthcare practitioners, researchers, and data and ethics experts, one significant strength of this study is the inclusivity and representation of different social groups, including disadvantaged or under-represented groups. Potential anticipated challenges include participant recruitment and generalisability of the study findings. This research will produce novel insights and knowledge to help inform policy and further research on the barriers and enablers to building trust and confidence in the access, sharing and use data for secondary purposes. The study will contribute to the debates and advance the methodology related to eliciting people’s views and perspectives in a safe, collaborative and efficient manner. Finally, the findings may be used to help promote a culture of trust that is mindful of peoples’ concerns about how their health data is used and shared.

## Data Availability

No underlying data are associated with this article
