# Supplementary material for: Building public trust and confidence in secondary use of health data for healthcare improvement and research: a qualitative study pre-protocol
Source: HRB Open Res. 2024 Jun 17;6:47. Originally published 2023 Sep 11. [Version 2] doi: 10.12688/hrbopenres.13711.2 (PMC11214037; doi:10.12688/hrbopenres.13711.2)
Supplement: Peer review history

# Reviewer report: Reviewer response for version 2

**DOI:** 10.21956/hrbopenres.15264.r40778

**Published:** 2024-06-28

**Authors:** Masoud Mohammadnezhad

**Affiliations:** 1 Birmingham City University, Birmingham, England, UK

Thank you for addressing all my comments.

Is the study design appropriate for the research question?

Yes

Is the rationale for, and objectives of, the study clearly described?

Yes

Are sufficient details of the methods provided to allow replication by others?

Yes

Are the datasets clearly presented in a useable and accessible format?

Not applicable

Reviewer Expertise:

Public Health Research, Qualitative and quantitative design, Social Determinants of Health and cross cultural research

I confirm that I have read this submission and believe that I have an appropriate level of expertise to confirm that it is of an acceptable scientific standard.

# Reviewer report: Reviewer response for version 1

**DOI:** 10.21956/hrbopenres.14997.r39425

**Published:** 2024-06-07

**Authors:** Masoud Mohammadnezhad

**Affiliations:** 1 Birmingham City University, Birmingham, England, UK

This is a good study that applied a qualitative cross-sectional approach to explore how to build public trust and confidence in the secondary use of health data through all key stakeholder groups in Ireland. It is a very relevant study that can help decision makers to support for secondary use of health data amongst the public, and an awareness of benefits for sharing health data. My decision to advise the authors to address these major comments.

 In the abstract and its method section- please put COREQ in the parenthesis.

 Please do the same in the method section of main text (put COREQ in the parenthesis).

 Explain the study setting. From where the study participants will be chosen and why?

 Please write all the inclusion and exclusion criteria. Do they need to be from Ireland? Will you exclude those who are not willing to participate? 

 PIn the abstract and its method section- please put COREQ in the parenthesis.

 Please do the same in the method section of main text (put COREQ in the parenthesis).

 Explain the study setting. From where the study participants will be chosen and why?

 Please write all the inclusion and exclusion criteria. Do they need to be from Ireland? Will you exclude those who are not willing to participate? 

 Please explain how did you measured the number of FGD? You mentioned one FGD from SG1-4 and 5 FGDs for the SG5.  How did you reach to this number?

 How did you develop the study tool/questionnaire? Any pilot study before applying in the main study?  

 Who will conduct interviews? Any previous experience?

 Why you easily change the FGD to in-depth interview for those who are not able to attend FGD. Don’t you think this may affect the quality of response?

 How about study trustworthiness? The credibility, transferability, dependability,..?

 lease explain how did you measured the number of FGD? You mentioned one FGD from SG1-4 and 5 FGDs for the SG5.  How did you reach to this number?

 How did you develop the study tool/questionnaire? Any pilot study before applying in the main study?  

 Who will conduct interviews? Any previous experience?

 Why you easily change the FGD to in-depth interview for those who are not able to attend FGD. Don’t you think this may affect the quality of response?

 How about study trustworthiness? The credibility, transferability, dependability,..?

Is the study design appropriate for the research question?

Yes

Is the rationale for, and objectives of, the study clearly described?

Yes

Are sufficient details of the methods provided to allow replication by others?

Yes

Are the datasets clearly presented in a useable and accessible format?

Not applicable

Reviewer Expertise:

Public Health Research, Qualitative and quantitative design, Social Determinants of Health and cross cultural research

I confirm that I have read this submission and believe that I have an appropriate level of expertise to confirm that it is of an acceptable scientific standard, however I have significant reservations, as outlined above.

# Response

**Published:** 2024-06-12

**Authors:** Tina Bedenik

**Affiliations:** School of Population Health, Royal College of Surgeons in Ireland, Dublin, Ireland

We wish to thank the reviewer for the feedback. We respond to each of the points below.

**In the abstract and its method section- please put COREQ in the parenthesis.**

**Response: **We have placed COREQ in the parenthesis.

**Please do the same in the method section of main text (put COREQ in the parenthesis).**

**Response: **We have placed COREQ in the parenthesis.

**Explain the study setting. From where the study participants will be chosen and why?**

**Response: **We added information regarding study setting in the Abstract/Background of the revised version, and this information is present in the ‘Study design’ section of the former version. The participants are chosen because of their prior knowledge, experiences and interest in secondary health data use, which we outlined in ‘Participants and recruitment’ section of the paper.

**Please write all the inclusion and exclusion criteria. Do they need to be from Ireland? Will you**

**exclude those who are not willing to participate?**

**Response: **Both inclusion and exclusion criteria have been outlined in the section ‘Participants and recruitment’:

 “Participants will be selected based on the following inclusion criteria: belonging to one of the five identified groups, experience with secondary health data use (applicable to SG1-4), and an interest in participating in the exploration of this topic regardless of their prior knowledge of secondary health data use (SG5). The study will only include participants aged 18+ years who are able to provide written, informed consent. The study will exclude those who cannot communicate fluently in English language.”

 We added that all participants will be recruited from Ireland in ‘Participants and recruitment’ section of the revised version to increase clarity.

**Please explain how did you measured the number of FGD? You mentioned one FGD from SG1-4 and 5 FGDs for the SG5. How did you reach to this number?**

**Response: **We decided on five study groups to facilitate a discussion among all relevant stakeholders in Ireland regarding secondary use of health data. In addition, the literature suggests that in qualitative studies 4–8 focus group discussions are sufficient to reach saturation (Hennink & Kaiser, 2022). We included this information in the revised version.

**How did you develop the study tool/questionnaire? Any pilot study before applying in the main**

**study?**

**Response: **We provided information on the topic guide and the pilot in the ‘Data collection’ section of the article:

 “The topic guide was informed by the literature review and it had been discussed with the RCSI PPI Panel, Industry Partner IQVIA Ireland and HIQA, IPPOSI and the HSE. The topic guide tailored for SG5 (Patients and Public) will be piloted in an online semi-structured interview with a female PPI Panel member, and the topic guide tailored for SG1-4 will be piloted in an online semi-structured interview with a qualitative researcher in the RCSI School of Population Health.”

**Who will conduct interviews? Any previous experience?**

**Response: **Information is provided in the article, under section ‘Research team and reflexivity’:

 “The focus groups and any semi-structured interviews will be organised and conducted by TB.”

 We added the information regarding researcher’s previous experience in the revised version.

**Why you easily change the FGD to in-depth interview for those who are not able to attend FGD.**

**Don’t you think this may affect the quality of response?**

**Response: **Thank you for the comment. We did not conduct any in-depth interviews, and all data is collected via focus group discussions. We have amended the article to reflect this more accurately.

**How about study trustworthiness? The credibility, transferability, dependability,..?**

**Response: **In order to increase trustworthiness and methodological rigor we outlined in the article that two members of the research team will independently code two focus group transcripts, and discuss the results. This is to ensure intercoder reliability, and we added this information in the revised version. We also outlined researchers’ positionality to identify and reduce any bias, and therefore enable a production of more trustworthy knowledge. Finally, we will also refer to and contrast with any published literature in the area when reporting the study findings, to increase the credibility, transferability and dependability.

# Reviewer report: Reviewer response for version 1

**DOI:** 10.21956/hrbopenres.14997.r36560

**Published:** 2023-10-31

**Authors:** Aileen Grant

**Affiliations:** 1 School of Nursing and Midwifery, Robert Gordon University, Aberdeen, UK

This is a well written protocol for a study which will provide important data about perspectives of sharing digital health data in Ireland. Given the lack of research in this area I think the findings will be important internationally. 

 It is not clear what organisations or people will be explored for data sharing. I assumed it was within health care organisations but this could be clearer. In the UK with post- pandemic health care access issues there is anecdotal evidence patients would like access to their records to help with the work of being a multimorbid patient. I think it would being an interesting dimension if patients were asked about this too.

 Apart from this everything was clear.

Is the study design appropriate for the research question?

Yes

Is the rationale for, and objectives of, the study clearly described?

Yes

Are sufficient details of the methods provided to allow replication by others?

Yes

Are the datasets clearly presented in a useable and accessible format?

Not applicable

Reviewer Expertise:

Qualitative methods

I confirm that I have read this submission and believe that I have an appropriate level of expertise to confirm that it is of an acceptable scientific standard.

# Response

**Published:** 2024-06-12

**Authors:** Tina Bedenik

**Affiliations:** School of Population Health, Royal College of Surgeons in Ireland, Dublin, Ireland

We would like to thank the reviewer for the helpful comment.

 We have outlined in the section ‘Participants and recruitment’ who are the people that will take part in the study: 

 Study Group 1 (SG1): Academics and Researchers using secondary data for research; 

 Study Group 2 (SG2): Healthcare Professionals from primary care and hospital settings; 

 Study Group 3 (SG3): Data Protection, Ethics and Privacy Experts and Data Controllers involved in health data; 

 Study Group 4 (SG4): Pharmaceutical Industry; 

 Study Group 5 (SG5): Patients and Public. 

 We added information that all participants will be recruited from Ireland, to increase clarity. 

 Thank you for the suggestion regarding the inclusion of perspectives of patients with multimorbidity. In the patients and public study group we include both persons with comorbidity and multimorbidity, and their views on the interactions with multiple healthcare providers and the impact on data sharing and use.
